# Experimental Study on Carbon Fiber-Reinforced Polymer Groove Machining by High-Power Water-Jet-Guided Laser

**DOI:** 10.3390/mi14091721

**Published:** 2023-08-31

**Authors:** Shuo Meng, Yugang Zhao, Dandan Zhao, Chuang Zhao, Yu Tang, Zhihao Li, Hanlin Yu, Guangxin Liu, Chen Cao, Jianbing Meng

**Affiliations:** School of Mechanical Engineering, Shandong University of Technology, Zibo 255049, China; m1369160775@126.com (S.M.); zhaodandansdut@126.com (D.Z.); zhaochuangsdut@126.com (C.Z.); tangyu1232022@126.com (Y.T.); lzhsdut@126.com (Z.L.); shuchenyhl@126.com (H.Y.); liuguangxin@sdut.edu.cn (G.L.); caochen@sdut.edu.cn (C.C.); jianbingmeng@sdut.edu.cn (J.M.)

**Keywords:** high-power water-jet-guided laser, RSM, CFRP, cutting depth

## Abstract

Due to the excellent properties of carbon fiber-reinforced polymers (CFRPs), such as high strength and strong corrosion resistance, the traditional water-jet-guided laser (WJGL) technology has problems with fiber pull-out and has a small cutting depth when processing CFRPs. Therefore, in this study, we used high-power water-jet-guided laser (HPWJGL) technology to perform groove processing experiments on CFRPs. The effects of four key process parameters, high laser power, pulse frequency, feed rate, and water-jet pressure, on the cutting depth were investigated by a single-factor experiment. The formation mechanism of groove cross-section morphology and the processing advantages of high-power water-jet-guided lasers were analyzed. On this basis, the mathematical prediction model of cutting depth was established by using the response surface method (RSM), and the optimal combination of process parameters was obtained. The mathematical prediction model was verified by experiments, and the error was only 1.84%, indicating that the model had a high reference value. This study provides a reference for the precision machining of HPWJGL technology.

## 1. Introduction

Carbon fiber-reinforced polymers (CFRPs) are widely used in various industries due to their excellent performance. CFRPs are an emerging composite material with epoxy resin as the matrix and carbon fiber as the reinforcing material. Because of its high stiffness, strength, and fatigue resistance, it is widely used in aircraft fuselage materials and sports car fluid shells [1]. Moreover, CFRPs also have relatively low density, a high damping capacity, good dimensional stability, and good corrosion resistance, so they are widely used in high-tech fields such as marine engineering, civil engineering, wind turbines, and robot engineering [2]. Due to the wide application of CFRPs, people are committed to exploring one of their most effective processing methods.

To meet the huge demand for CFRPs in various industries, the method of processing CFRPs has become a hot spot in current machining. Because CFRPs are composed of two different materials (carbon fiber and epoxy resin), traditional contact machining such as drilling, sawing, and wire cutting often causes problems such as serious tool wear, fiber pull-out, and interlayer cracks [3]. Among them, stratification is the main threat to the loss of mechanical strength and fatigue strength in the drilling area [4]. At present, the common methods for processing CFRPs are abrasive water-jet processing, ultrasonic vibration-assisted (UVA) processing, and electrical discharge machining (EDM). Abrasive water-jet machining uses a high-pressure water-jet to drive abrasive particles into workpieces. The advantage of this machining method is that the thermal damage is small, but the epoxy resin matrix will become softer as the humidity increases, resulting in poor adhesion of the fiber matrix and then processing defects such as notch deformation, cone angle formation, and material delamination [5]. Machining accuracy cannot be guaranteed. Ultrasonic vibration-assisted machining is a process that uses micro-scale high-frequency vibration applied to cutting tools to improve material removal efficiency. When machining CFRPs, problems such as excessive tool wear and subsurface damage to the workpiece will occur [6,7]. The principle of electrical discharge machining is that a series of voltage pulses are transmitted between the electrode and the workpiece to generate an electric spark, and the high temperature generated by the electric spark is used to remove excess material from the workpiece [8]. However, the high current density during processing leads to the coating of epoxy resin on the surface, which reduces the material removal rate and causes rapid deterioration of the machined surface [9]. As a new processing method with no contact, no tool wear, and clean energy, laser processing stands out among mechanical processing methods. However, due to the large difference in the thermal properties of the two composite materials of CFRPs, the thermal damage to the material during the processing will be large, and due to the high thermal conductivity of the carbon fiber, the thermal energy will diffuse along the carbon fiber to the material, which will lead to further expansion of the heat-affected zone [10,11]. Riveiro studied the influence of processing parameters of CO_2_ laser cutting CFRPs on laser cutting efficiency and cutting quality. The results show that the heat-affected zone of laser processing is inevitable, but it can be reduced by selecting appropriate process parameters to obtain better processing quality [12]. Therefore, more and more people are studying how to reduce the thermal impact caused by laser processing to achieve high-precision processing of CFRPs.

In 1854, Tyndall found the total reflection of light in flowing liquid through experiments, which proved that a water-jet can be used as a medium to transmit light, which provides a theoretical basis for WJGL technology [13]. In 1991, Richerzhagen of the Swiss Federal University of Technology used a nozzle structure to generate a jet and focused the laser on the nozzle port, further enriching the principle of WJGL technology. Based on this research, he successfully developed a more perfect WJGL technology method in 1997 [14]. This new processing method adds a water-jet based on laser processing. While using the laser energy beam to remove the material, the water-jet can play a role in cooling and removing the slag from the material. This new processing method has become popular for precision machining because it has a small thermal impact and keeps the surface of the material clean after processing. Therefore, many scholars, both domestic and abroad, have studied this. HOCK uses a 532 nm laser to make an experimental comparison between traditional laser processing and WJGL-processing of brass and stainless steel sheets. The results show that although traditional laser processing has high processing efficiency, there is still a thick deposition layer and a large heat-affected zone in the processing area, while the WJGL processing area has no residue, the notch width is small, and there is almost no heat-affected zone [15]. Wagner used traditional laser processing technology and WJGL processing technology to cut metal sheets for experiments. The experimental results show that under the same cutting speed and processing efficiency, there is no burr or heat-affected zone on the surface processed by WJGL technology due to the scouring and cooling effect of the water-jet in the pulse gap [16]. Dong Sun studied the effect of WJGL cutting experiments at low power on the surface integrity of CFRPs. The experimental results show that the WJGL cutting CFRPs under low power can obtain better surface morphology and a smaller heat-affected zone, but the processing efficiency is low. Choosing a high-power WJGL is the main method to improve processing efficiency [17]. In summary, compared with the problems of thermal damage and thermal ablation caused by traditional laser processing, WJGL technology has absolute advantages in realizing non-thermal damage processing, but too small a laser power will affect the processing efficiency. Therefore, in this paper, we designed experiments to study the effect of high-power WJGL-processing CFRPs.

To study the feasibility of HPWJGL-processing CFRPs and solve the problem of low efficiency of low-power WJGL processing, a single-factor experiment for HPWJGL-cutting CFRPs was designed in this paper. The effects of high laser power, feed speed, pulse frequency, and water-jet pressure on cutting depth and heat-affected zones were analyzed through experimental results. Finally, a mathematical prediction model was established by the response surface method (RSM) and verified by experiments, and then the optimization parameters of high-power WJGL technology for CFRP processing with a high reference value were obtained. The theoretical and experimental results of this experiment provide a basis for the high-quality processing of CFRPs.

## 2. Experimental Principle

**Figure 1:**
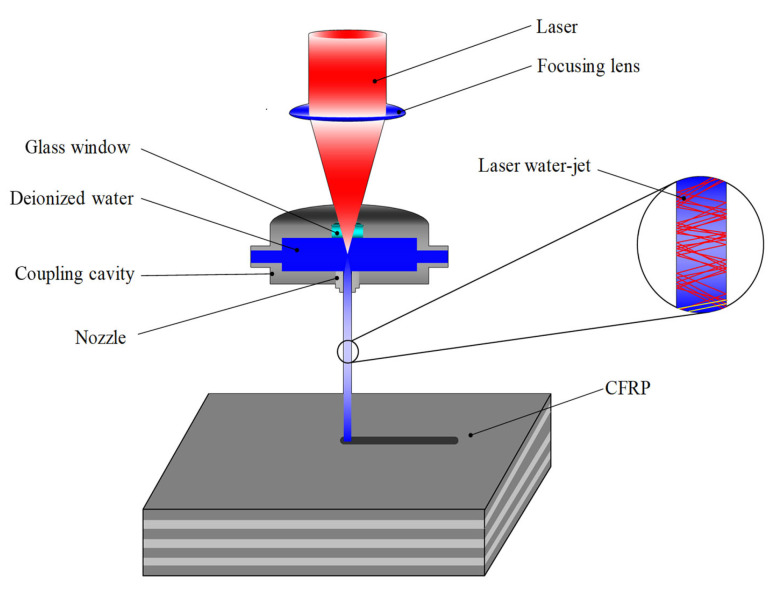
The principle of HPWJGL technology processing.

The principle of HPWJGL technology processing is shown in Figure 1. Because the refractive index of water and air is different, when the incident angle of the laser is less than the critical angle of total reflection, the laser beam will have a total reflection at the boundary between water and air. The laser beam is not directly focused on the surface of the workpiece but is transmitted to the surface of the workpiece through total reflection. This processing method is different from traditional water-jet processing, in which the high-pressure water-jet directly impacts and crushes the material. The water pressure in HPWJGL processing is low, and its main function is to carry away the molten material and excess heat while conducting the laser to the surface of the workpiece for processing [18]. Because the thermal and physical characteristics of carbon fiber and the epoxy resin matrix in CFRPs are different, there are different reactions in water-guided laser processing. The water-jet has the effect of scouring and cooling during processing, which can quickly take away the heat of the processing area and the residue, such as epoxy resin, that melts to the melting point. The carbon fiber will sublimate when it reaches the sublimation temperature, so the HPWJGL-processing of CFRPs will not result in the common edge irregularities and burrs caused by traditional laser processing. The high-speed water-jet of the water-conducting laser plays a role in cooling the processing area during the laser pulse gap, which can effectively reduce the heat-affected zone, reduce the thermal deformation and thermal damage of the material, and improve processing accuracy.

## 3. Experiment Setup

### 3.1. Experiment Equipment

**Figure 2:**
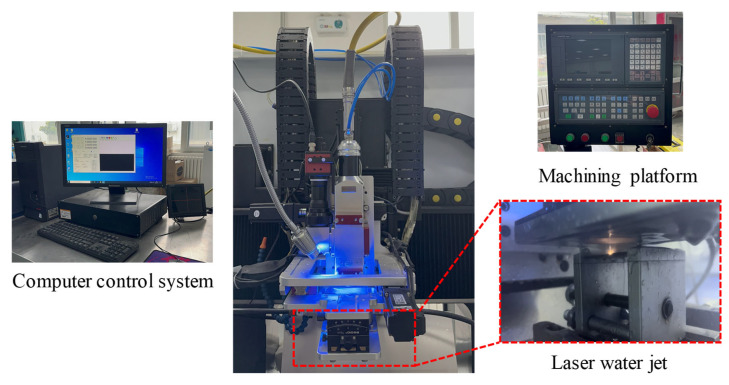
HPWJGL processing equipment.

The self-built HPWJGL processing equipment used in this experiment is shown in Figure 2. The equipment consists of a water–light coupling alignment system, a water supply system, and a motion platform. The water–light coupling alignment system is composed of a YLR-2000-WC (DSX1000, OLYMPUS, Tokyo, Japan) fiber laser, beam focusing element, and camera. The maximum power of the fiber laser is 2000 W, the wavelength is 1070 nm, and the maximum laser repetition frequency is 10 kHz. In this experiment, the diameter of the nozzle is 0.5 mm, the distance between the nozzle and the workpiece is 15 mm, and the effective diameter in contact with the workpiece is 0.5 mm. During the experiment, the microparticles and bubbles in the water interfered with the laminar flow state of the jet and even lead to a change in the optical transmission path. Therefore, the water supply system used in this experiment consists of a high-pressure precision filter, a three-cylinder plunger pump, a pressure-regulating valve, and an overflow valve. The maximum water pressure is 5 MPa, which can produce filtered deionized water and ensure better processing quality.

### 3.2. Experimental Design

**Figure 3:**
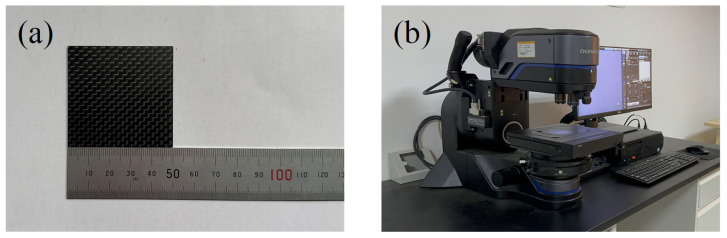
(**a**) Experimental plate and (**b**) DSX ultra-depth-of-field microscope equipment.

In this experiment, 50 × 50 × 1.5 mm orthogonally distributed epoxy resin-based carbon fiber experimental plates were selected. The experimental plate is shown in Figure 3a. The physical properties of CFRPs at room temperature are shown in Table 1. To determine the appropriate range of the main parameters of HPWJGL technology for machining CFRPs, a single-factor experiment was designed to study the influence of laser power, laser frequency, laser pulse, and feed rate on the cutting depth of CFRPs. To obtain better cutting depth and groove surface morphology, based on reading relevant literature and a large number of experiments, the parameters of the HPWJGL-processing CFRP experiment were selected as follows: laser power 100~300 W, pulse frequency 3000~7000 Hz, water pressure 0.4~1.2 MPa, and feed speed 0.1~0.3 mm/s. The experiment used a single-cutting experiment with four factors and five levels. Each group of experiments was repeated three times, and the average of the three was taken. After the processing was completed, the surface of the plate was cleaned with anhydrous ethanol and then further cleaned with an ultrasonic cleaner. Finally, a DSX ultra-depth-of-field microscope was used to obtain a clear image of the gully surface of the CFRP specimen and measure the cutting depth. The DSX ultra-depth-of-field microscope equipment is shown in Figure 3b.

## 4. Single-Factor Experiment Design and Result Analysis

### 4.1. Influence of High Laser Power on Cutting Quality

**Figure 4:**
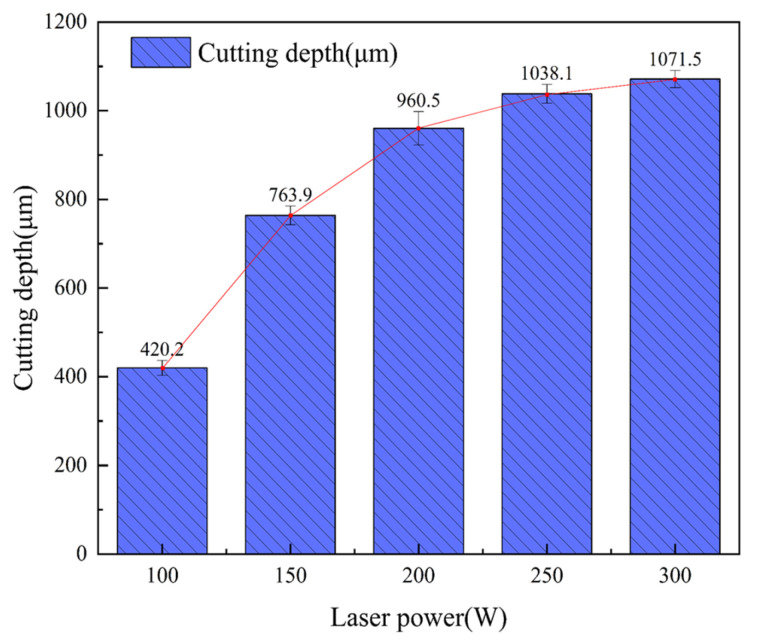
The average cutting depth at different laser power.

**Figure 5:**
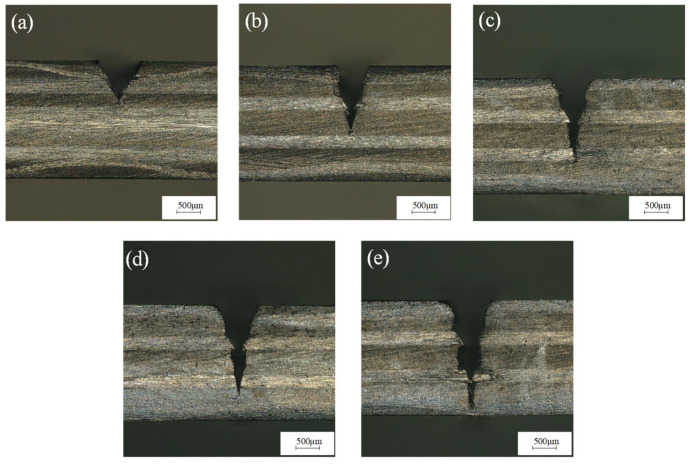
Morphology of grooves section at (**a**) 100 W, (**b**) 150 W, (**c**) 200 W, (**d**) 250 W, and (**e**) 300 W laser power.

The feed speed was 0.2 mm/s, the water pressure was 0.8 MPa, the laser frequency was 6000 Hz, and the laser power was 100 W, 150 W, 200 W, 250 W, and 300 W for single-cutting experiments, respectively. Figure 4 shows the average cutting depth at different laser powers. It can be seen from Figure 4 that the cutting depth gradually increases with an increase in laser power, but the increment gradually decreases. The reason is as follows: when the laser frequency and pulse width are constant, the single-pulse energy is determined by the laser power and increases with the increase of the laser power. The removal ability increases with the increase in pulse energy, so that the material reaching the removal threshold gradually increases, which makes the cutting depth increase. Figure 5 shows the effect of high laser power on the groove cross-section morphology. It can be seen from the figure that the cutting cross-section is generally V-shaped. This is because the energy density of the laser center in the water-jet-guided laser is large and the water pressure at the center of the water-jet is large. Because the V-shaped notch gradually narrows from top to bottom, it will lead to difficulty in slag discharge, so the laser energy cannot reach the lower end of the groove smoothly, and the energy transmitted to the side wall of the groove increases, thus further expanding the V-shaped notch. In the case of a 90° arrangement, the fibers in the composite material are filled with resin, and the heat will be transferred alternately between the fibers and the resin, so the heat is not easily spread in this direction, so the width of the transverse and longitudinal distribution of the carbon fiber is different. Figure 5a,b show the cross-section morphology of the groove under laser powers of 100 W and 150 W, respectively. Due to the low laser power and the absorption of laser energy by the water-jet, the laser cannot cut the carbon fiber wire in time, resulting in low cutting depth and some problems such as fiber exposure and pull-out. Figure 5c,d show the groove section under the laser power of 200 W and 250 W, respectively. There are some carbon fiber filaments exposed in the groove section, but the heat-affected zone is small, no carbon fiber filaments are pulled out, and the groove section morphology is better. In Figure 5e, the laser power is 300 W, the cutting depth increases slowly compared with the low power, the heat-affected zone increases, and some carbon fiber filaments are brittle and pulled out. This is because when the laser power is too large, the energy transmitted to the inner wall of the groove increases, resulting in an increase in the heat-affected zone and the width of the incision. Therefore, when other parameters remain unchanged when the power is in the range of 200~300 W, the cross-section morphology of the groove obtained by processing is better.

### 4.2. The Influence of Feed Speed on Cutting Quality

**Figure 6:**
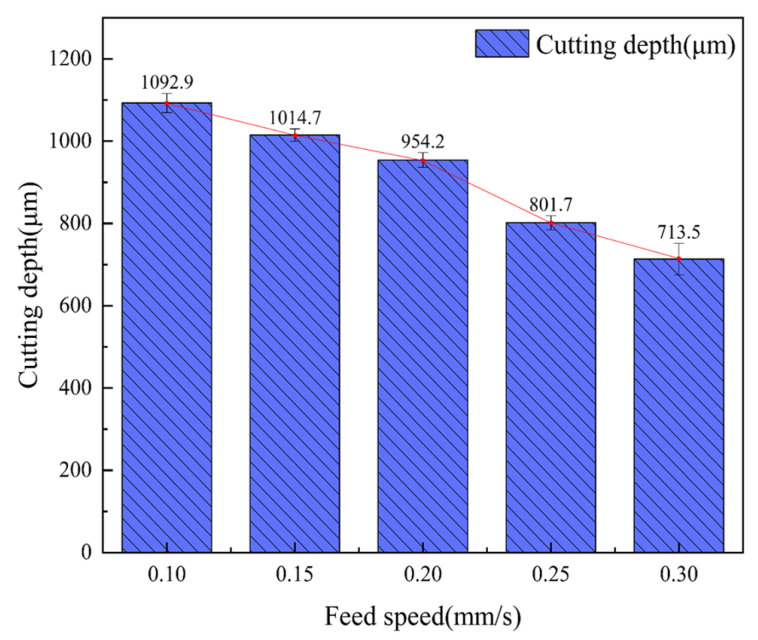
The average cutting depth at different feed speeds.

**Figure 7:**
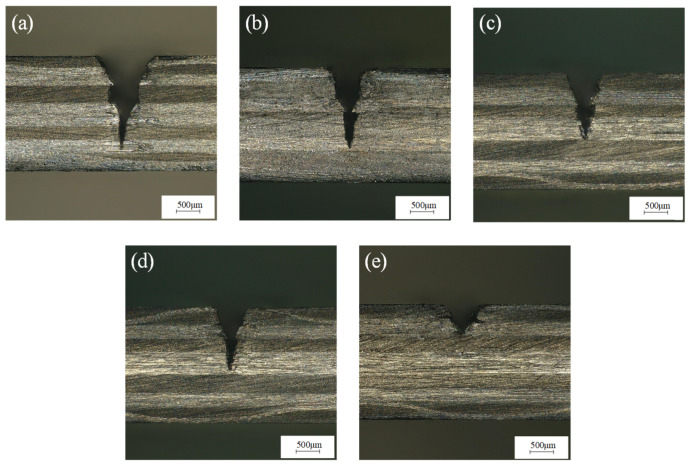
Morphology of grooves section at (**a**) 0.1 mm/s, (**b**) 0.15 mm/s, (**c**) 0.2 mm/s, (**d**) 0.25 mm/s, and (**e**) 0.30 mm/s feed speed.

The laser frequency was 6000 Hz, the water-jet pressure was 0.8 MPa, the laser power was 200 W, and the feed speed was 0.1 mm/s, 0.15 mm/s, 0.2 mm/s, 0.25 mm/s, and 0.3 mm/s for single-cutting experiments, respectively. Figure 6 shows that the cutting depth decreases with an increase in feed speed. This is because with the increase in feed speed, the number of pulses of laser radiation in the same area decreases, the interaction time between the water-jet-carrying laser and the CFRP material decreases, and the laser pulse energy absorbed by the CFRP material per unit area decreases, resulting in a decrease in cutting depth. Figure 7a shows the groove interface morphology at a feed speed of 0.1 mm/s. It can be seen from the figure that the material can absorb enough laser energy at a feed speed of 0.1 mm/s, so the obtained notch width and cutting depth are larger. And because the laser pulse energy absorbed by the CFRP material per unit area is higher, the energy transmitted to the inside of the groove is also increased, which leads to a larger heat-affected zone and is accompanied by fiber exposure. Figure 7b,c show the cross-section morphology of the groove at a feed speed of 0.15 mm/s and 0.2 mm/s, respectively. The heat-affected zone is small, and no fiber pull-out occurs. Figure 7d,e show the cross-section morphologies of grooves at feed speeds of 0.25 mm/s and 0.3 mm/s, respectively. It can be seen from the figure that when the feed speed is greater than 0.2 mm/s, the cutting depth is small. The reason is that with the increase in feed speed, the laser pulse energy absorbed by the CFRP material per unit area decreases, the cutting depth decreases, and the fiber pull-out phenomenon exists. Therefore, when the feed speed is in the range of 0.1~0.2 mm/s, the machining quality of the groove section is better.

### 4.3. Influence of Laser Pulse Frequency on Cutting Quality

**Figure 8:**
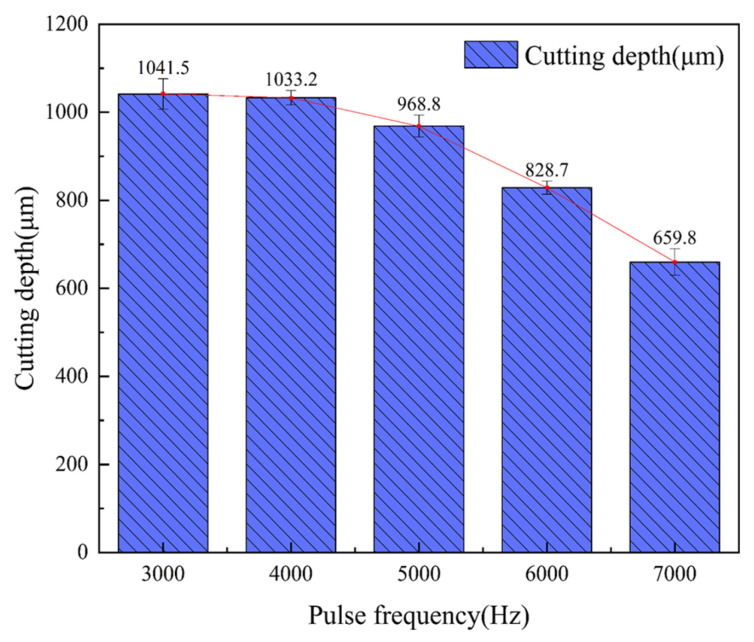
The average cutting depth at different pulse frequencies.

**Figure 9:**
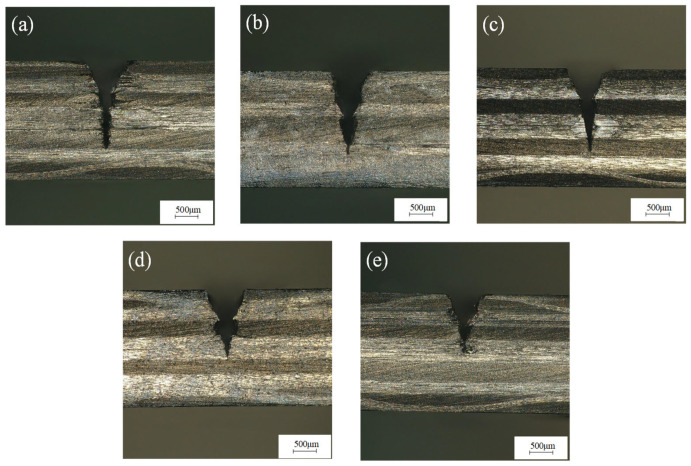
Morphology of grooves section at (**a**) 3000 Hz, (**b**) 4000 Hz, (**c**) 5000 Hz, (**d**) 6000 Hz, and (**e**) 7000 Hz pulse frequency.

The laser power was 200 W, the water-jet pressure was 0.8 MPa, the feed rate was 0.2 mm/s, and the laser pulse frequencies were 3000 Hz, 4000 Hz, 5000 Hz, 6000 Hz, and 7000 Hz for single-cutting experiments, respectively. Figure 8 shows that with the increase in laser repetition frequency, the incision width decreases and the cutting depth decreases rapidly. The reason is that when the laser power is constant, with the increase in laser repetition frequency, the single-pulse energy of the laser decreases significantly, the laser removal ability decreases, and the cutting depth decreases. Figure 9a shows the groove cross-section morphology at the laser pulse frequency of 3000 Hz. It can be seen from Figure 9 that the cutting depth is larger, but the single-pulse energy is larger, and the laser pulse duty cycle is smaller, resulting in a larger heat-affected zone of the material. Figure 9b–d show the cross-section morphology of the groove under the laser pulse frequencies of 4000 Hz, 5000 Hz, and 6000 Hz, respectively. The heat-affected zone of the groove section is small, there is no fiber pull-out, and the processing morphology is good. It can be seen from the cross-section morphology of the groove at the laser pulse frequency of 7000 Hz shown in Figure 9e that there is a small cutting depth and a large heat-affected zone. This is because when the laser pulse frequency is greater than 6000, the removal ability of the laser pulse is very weak, and because of that, some carbon fibers cannot be cut off, which hinders the downward transmission of laser energy, resulting in a decrease in ablation depth and ablation width. Moreover, the duty cycle of the laser pulse is large, which is not conducive to the heat dissipation of the material, resulting in more heat transfer to the inside of the groove, resulting in a larger heat-affected zone. Therefore, when the pulse frequency is between 4000 Hz and 6000 Hz, the processing quality of the groove section is the best.

### 4.4. Influence of Water Pressure on Cutting Quality

**Figure 10:**
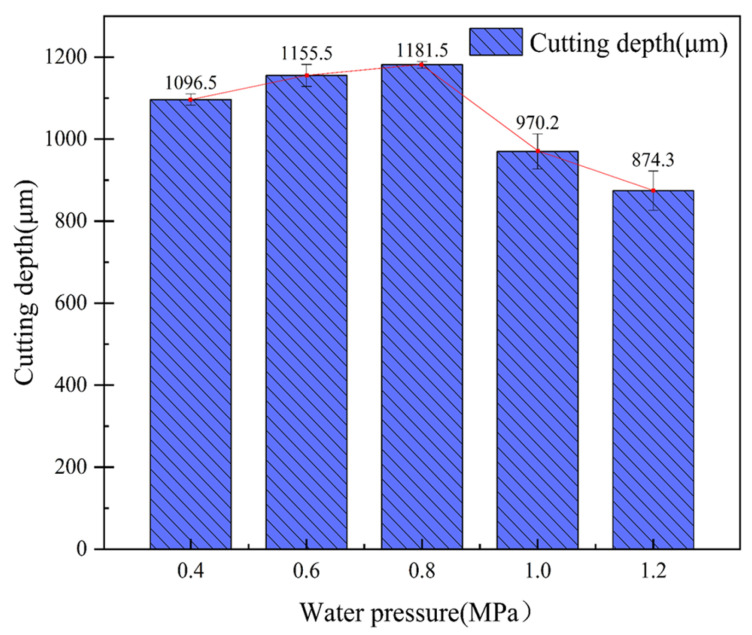
The average cutting depth at a different water pressure.

**Figure 11:**
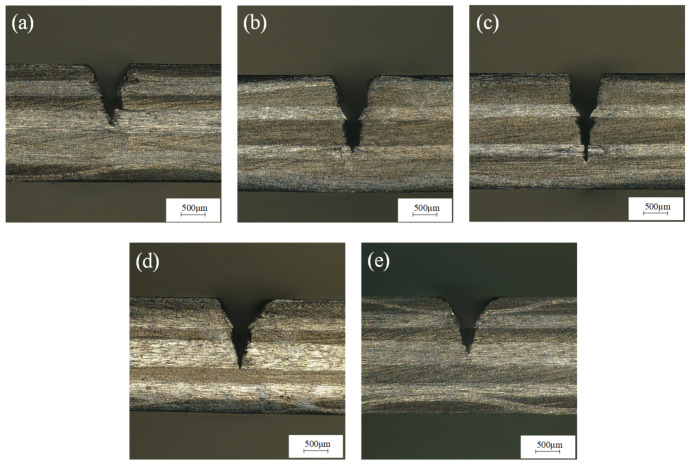
Morphology of grooves section at (**a**) 0.4 MPa, (**b**) 0.6 MPa, (**c**) 0.8 MPa, (**d**) 1.0 MPa, and (**e**) 1.2 MPa water pressure.

The laser power was 200 W, the laser frequency was 6000 Hz, the feed rate was 0.2 mm/s, and the water-jet pressure was 0.4 MPa, 0.6 MPa, 0.8 MPa, 1.0 MPa, and 1.2 MPa for a single-cutting experiment, respectively. It can be seen from Figure 10 that when other parameters remain unchanged, the cutting depth increases first and then decreases with the increase in water pressure. Figure 11a shows the cross-section morphology of the groove under a water pressure of 0.4 MPa. It can be seen from Figure 11a that the heat-affected zone is large, and there are problems with fiber embrittlement and fiber exposure. This is because when the water pressure is small, the cooling effect of the water-jet is less than the laser energy absorbed by the CFRP, and the molten material generated by the CFRP, which absorbs the laser pulse energy, cannot be washed away in time. Figure 11b,c show the cross-sectional morphologies of the groove’s underwater pressures of 0.6 MPa and 0.8 MPa, respectively. The cutting depth shows an increasing trend, the heat-affected zone is small, and there is almost no problem with fiber pull-out or exposure. This is because, with the increase in water pressure, the velocity of the water-jet increases, the scouring ability of the water-jet increases, and the molten material can be taken away by the water-jet in time, which does not affect the downward transmission of the laser or the absorption of laser energy by materials. Therefore, the cutting depth increases with the increase in water-jet pressure, and the heat-affected zone also shows a downward trend. With the increase in water-jet pressure, the shrinkage effect of the water-jet gradually increases, and the diameter of the water-jet gradually decreases. Because of that, the slit width decreases. Figure 11d,e show the cross-section morphology of the groove under water pressures of 1.0 MPa and 1.2 MPa, respectively. When the water-jet pressure is greater than 0.8 MPa, the stability of the water-jet beam becomes worse, resulting in a decrease in the laser energy transmitted to the CFRP material and a decrease in the cutting depth. Because the epoxy resin matrix on the surface of the groove is easily decomposed by heat, with the increase in water-jet pressure, the erosion effect of the water-jet on the surface of the groove will be more obvious, increasing the width of the incision. Therefore, when other parameters remain unchanged when the water-jet pressure is between 0.6 MPa and 0.8 MPa, the processing quality of the groove section is better.

## 5. Analysis of Response Surface Experimental Results

### 5.1. Regression Model and Variance Analysis

The design of the experiment (DOE) is a systematic method for allocating the correlation between input and output responses. The aim is to use DOE to collect experimental results for verification and statistical methods for analysis. In this paper, the response surface method (RSM) was used for experimental design and analysis. RSM can fit the nonlinear function relationship between the input factor and the response quantity by establishing a multivariate quadratic regression equation model to select the best process scheme within the known optimization range. The optimization process scheme of this paper is based on the experimental range and measurement results obtained under the single-factor experiment. The RSM is used to optimize the process parameters of HPWJGL technology for cutting CFRPs, and the optimal values of the four process parameters of laser power, pulse frequency, feed speed, and water-jet pressure are further obtained.

The Box–Behnken module in the Design-Expert software (DX10) was used for the experimental design. The process parameters of HPWJGL are selected: laser power 200~300 W, pulse frequency 4000~6000 Hz, feed rate 0.1~0.2 mm/s, and water-jet pressure 0.6~0.8 MPa. A four-factor, three-level response surface test was designed with a total of 29 groups of experiments, of which five groups were repeated under the same factor to estimate the main effects and interactions of each factor [19]. The advantage is that the relative error of the test is reduced, and the experimental results are more accurate. The factor level table is arranged as shown in Table 2.

The Box–Behnken module in the Design-Expert software was used to design the experiment. Taking the cutting depth as the index, three sets of experiments were carried out at each test point, and the average value was calculated. The response surface experimental design and results are shown in Table 3. The regression equation of cutting depth is obtained by fitting the experimental data:

$$\begin{array}{l} H=1106.98+56.70\times A-40.82\times B-36.27\times C+22.63\times D+22.45\times \mathrm{AB}-2.65\times \mathrm{AC}-17.35\times \mathrm{AD}\\ -10.30\times \mathrm{BC}+14.95\times \mathrm{BD}+19.20\times \mathrm{CD}-19.64\times A^{2}-0.39\times B^{2}-22.64\times C^{2}+0.060\times D^{2} \end{array}$$

The results of the quadratic model analysis of variance and F test of the CFRP processed by HPWJGL technology are shown in Table 4. The F-value is the ratio of the mean square between the groups and the mean square within the group, and the p-value is the probability value corresponding to the F-value. If the *p*-value is >0.1, it is not significant [19]. The F-value of the model selected in this experiment is 31.84, and the *p*-value is less than 0.0001, indicating that the established response surface regression equation prediction model of the four independent variables affecting the cutting depth is very significant, so the model can effectively predict the relationship between the four independent variables and the cutting depth. The *p*-value of the fitted missing term is 0.7243 > 0.05, indicating that the regression equation of the fitted missing term is not significant. The multivariate correlation coefficient R^2^ = 0.9696. After correction, the multivariate correlation coefficient Adj R^2^ = 0.9391, indicating that the incision depth regression model can explain 93.91% of the response value, indicating that the actual error is small and the fitting degree is good. Among the four factors that affect the cutting depth, the *p*-values of laser power, pulse frequency, feed speed, and water-jet pressure are all <0.0001, indicating that the laser power, pulse frequency, feed speed, and water-jet pressure are highly fitted with the cutting depth. The F-value of laser power is 182.73, which indicates that laser power has the greatest influence on the cutting depth. The reason is that laser power is the main energy source in the processing of HPWJGL. The larger the laser power, the greater the energy density of the laser, and a larger surface volume of the material is removed. Under the erosion of the water-jet, the molten material is discharged, thus forming a deeper groove.

**Figure 12:**
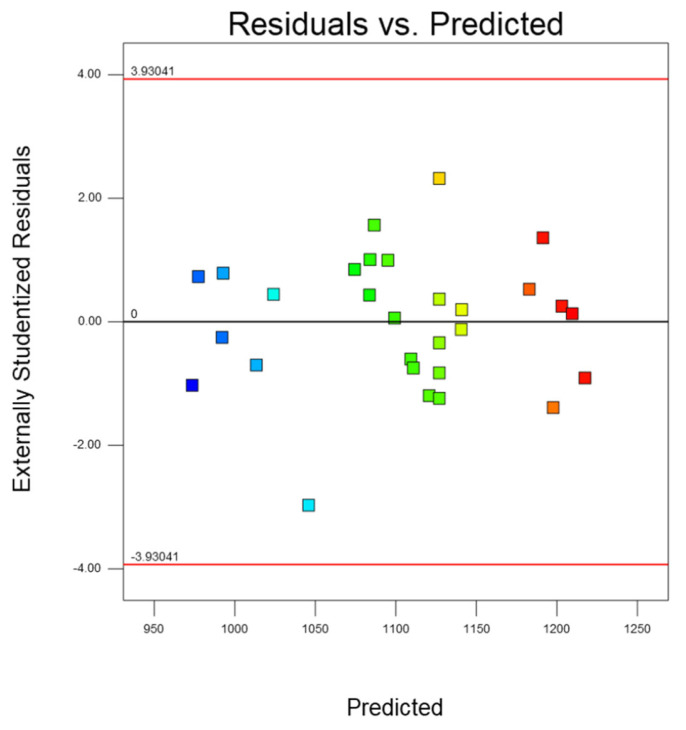
Relationship between residual error and predicted value.

**Figure 13:**
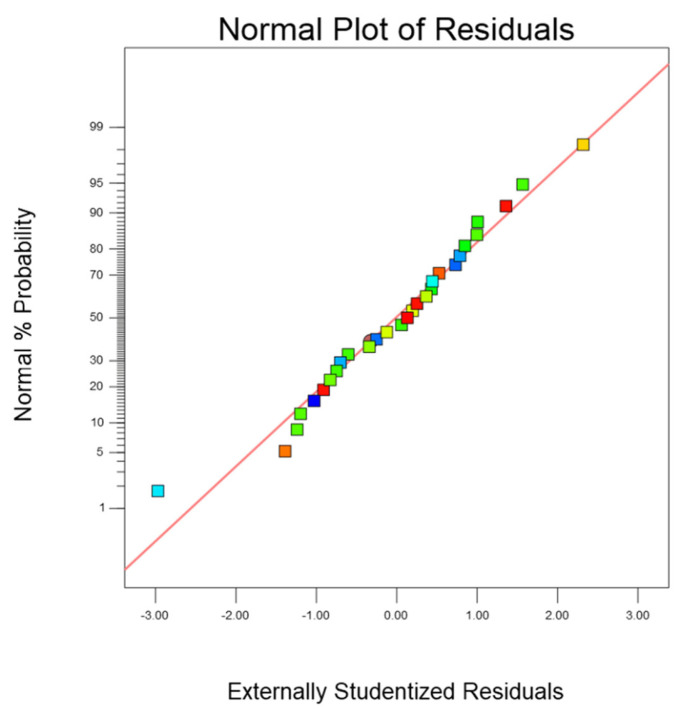
Normal distribution diagram of residuals.

The fitting of the model can be evaluated by the normal graph of the residual value and the figure of the relationship between the residual and the predicted value. Figure 12 shows that the data are scattered around the average line, indicating that the established model is statistically significant. Figure 13 is the residual normal distribution diagram of the prediction model, which can be used to test the advantages and disadvantages of the model. It can be seen from Figure 13 that the normal distribution of the model is evenly distributed around the straight line, which is highly fitted to the straight line. Therefore, it can be judged that the predicted value of the model is in good agreement with the actual value.

### 5.2. Response Surface Analysis of Process Parameters

#### 5.2.1. Response Surface Analysis of Laser Power and Feed Speed

**Figure 14:**
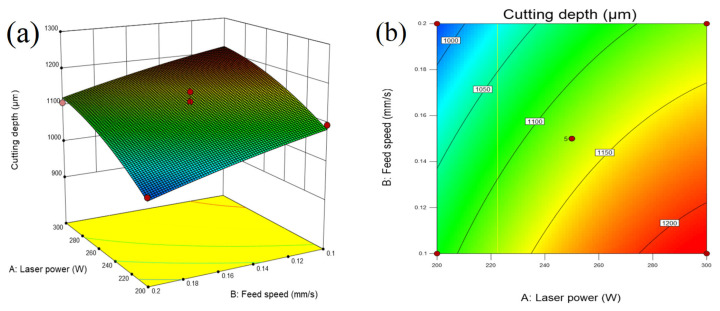
(**a**) Three-dimensional surface diagram and (**b**) contour map of the influence of laser power and feed speed on cutting depth.

The three-dimensional surface map and contour map of the response can directly reflect whether the interaction between the two variables has a significant effect on the response value. Figure 14a,b show that when the pulse frequency and water pressure are fixed, the cutting depth increases with the increase in laser power and decreases with the decrease in cutting speed.

#### 5.2.2. Response Surface Analysis of Laser Power and Pulse Frequency

**Figure 15:**
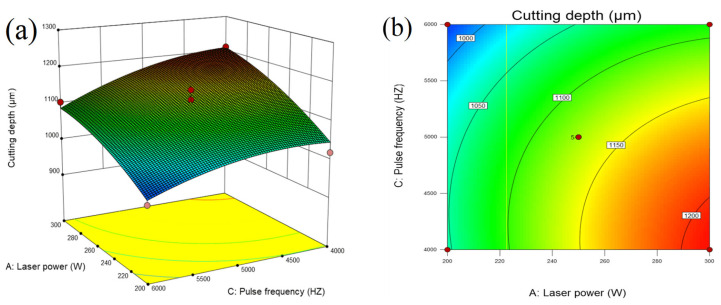
(**a**) Three-dimensional surface diagram and (**b**) contour map of the influence of laser power and pulse frequency on cutting depth.

**Figure 16:**
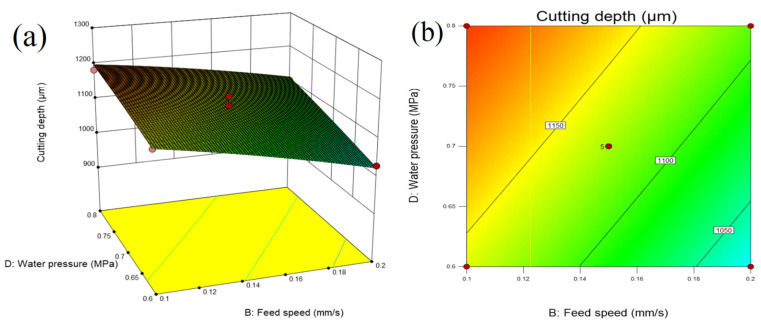
(**a**) Three-dimensional surface diagram and (**b**) contour map of the influence of feed speed and water pressure on cutting depth.

Figure 15a and Figure 16 show that when the feed speed and water pressure are fixed, high laser power and a low repetition rate are beneficial to increasing the cutting depth. The reason is that with the increase in laser power, the decrease in pulse frequency, and the increase in single-pulse energy, the stronger the ability to remove materials is, therefore the cutting depth increases [20]. However, there will still be the problem of a too large heat-affected zone. Therefore, in order to ensure processing quality, the laser power should be increased and the pulse frequency should be reduced as much as possible.

#### 5.2.3. Response Surface Analysis of Feed Speed and Water Pressure

Figure 16 shows that when the laser power and pulse frequency are constant, the cutting depth increases with the increase in water pressure at a lower feed speed. The reason is related to the stability of the water-jet. When the water pressure is low, the water-jet velocity is low, and the molten material formed by the water-jet-guided laser cutting cannot be eliminated in time, so the laser energy cannot continue to transmit downward, resulting in a smaller cutting depth. After the water-jet enters the nozzle orifice, the water beam ejected has a shrinkage phenomenon, and the shrinkage range is about 83% of the nozzle diameter [21]. When the water-jet pressure is large, the formed water beam has a good shrinkage effect, and the water flow is relatively stable. The laser energy reaching the surface of the workpiece through water–light coupling increases, and the molten material generated by laser cutting can also be removed in time, so the cutting depth is high. Therefore, in the stable range of the water-jet, a higher water-jet pressure should be selected.

### 5.3. Process Parameter Optimization and Experimental Verification

Using the Design-Expert software to predict and optimize the process parameters of HPWJGL technology for cutting the CFRP with the maximum cutting depth as the optimization goal, the process parameters of HPWJGL technology for cutting the CFRP are predicted and optimized. The results show that when the laser power is 271.49 W, the feed rate is 0.149 mm/s, the pulse frequency is 4048.24 Hz, and the water-jet pressure is 0.799 MPa, the predicted cutting depth is the best, which is 1212.257 μm.

**Figure 17:**
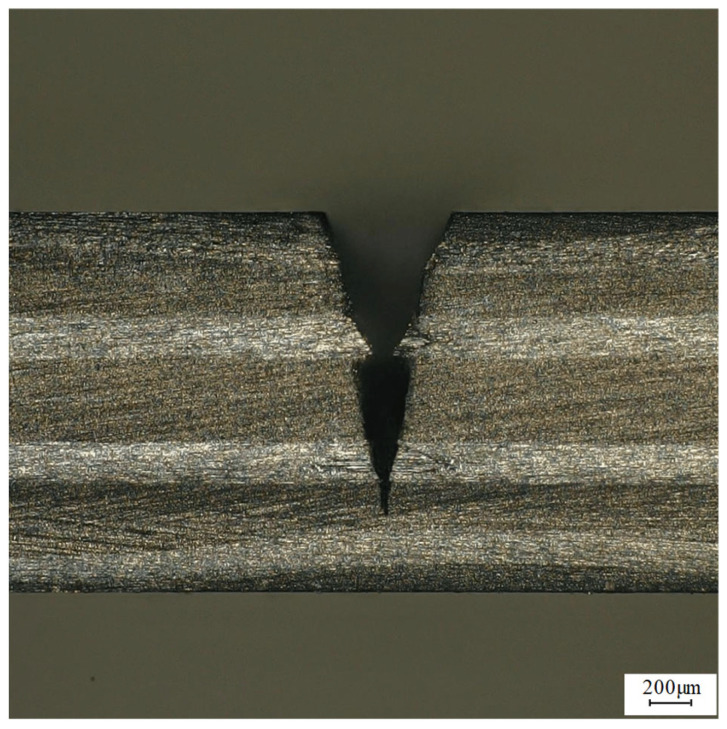
The optimized groove section morphology diagram.

To verify the reliability of the response surface optimization method, cutting experiments were carried out using the optimized process parameters. Three sets of experiments were carried out, and the average value was obtained. The test results are shown in Table 5. The cutting section is shown in Figure 17. The groove section morphology obtained by using the optimized process parameters is better, the heat-affected zone is smaller, and there is a slight problem of fiber exposure, but there is no phenomenon such as fiber pull-out. The actual measurement depth is 1189.97, and the error with the predicted value is 1.84%. The error is small, indicating that the prediction model has a high reference value. The reason for this error may be a system error in the experimental equipment or an error in the measurement process.

## 6. Conclusions

In this paper, the HPWJGL technology for processing CFRPs was studied. The influence of different process parameters on the machining effect was investigated by a single-factor experiment, and the mathematical model of optimizing the cutting depth was established by the response surface method. According to the experimental results and model predictions, the following conclusions are drawn:

1. The single-factor test results show that the laser power, feed rate, pulse frequency, and water-jet pressure have a significant effect on the cutting depth. However, improper selection of process parameters will cause adverse effects such as a too large heat-affected zone. In the experiment of water-guided laser cutting of CFRPs, the laser power determines the energy of a single pulse, so it has the greatest influence on the cutting depth. The experimental results show that when the laser power is 200~300 W, the feed speed is 0.1~0.2 mm/s, the laser frequency is 4000~6000 Hz, and the water-jet pressure is 0.6~0.8 MPa, the cutting effect is promoted.
2. The response surface test scheme of four factors and three levels was developed with the cutting depth as the evaluation standard by the response surface method. The regression model of the influence of laser power, feed rate, pulse frequency, and water-jet pressure on the cutting depth is established. The results show that the model has a response value of 93.91%, and its good fitting effect is verified by variance analysis, which has a high reference value. In addition, the level of influence of each of the parameters on the cutting depth is laser power > pulse frequency > feed rate > water-jet pressure.
3. The response surface optimization results show that the process parameters are a laser power of 271.49 W, a feed rate of 0.149 mm/s, a pulse frequency of 4048.24 Hz, a water-jet pressure of 0.799 MPa, and a predicted depth of 1212.257 mm. Finally, the cutting experiment is carried out by using the optimized parameters to verify the predicted value. The cutting depth is 1189.97 mm, and the error is only 1.84%, which proves that the model has a high reference value.
4. There are many factors affecting the quality of water-guided laser processing. In this paper, only some process parameters are selected to explore the processing quality. Subsequently, water-jet diameter, processing distance, CFRP material thickness, and other factors can be selected for further experimental research.

## Figures and Tables

**Table 1:** Physical properties of the CFRP at room temperature.

Density	Melting Temperature	Thermal Conductivity
Kg/m^3^	°C	(W/(m·K))
1500 ± 15	3300	32(0°), 5(90°)

**Table 2:** Box–Behnken design test factors and levels.

Test Factors	Laser Power (A)	Feed Speed (B)	Pulse Frequency (C)	Water Pressure (D)
Unit	W	mm/s	Hz	MPa
1	200	0.1	4000	0.6
2	250	0.15	5000	0.7
3	300	0.2	6000	0.8

**Table 3:** The response surface experimental design and results.

Test Factors	Laser Power (A)	Feed Speed (B)	Pulse Frequency (C)	Water Pressure (D)	Cutting Depth (H)
Unit	W	mm/s	Hz	MPa	μm
1	200	4000	0.15	0.7	1018.6
2	250	5000	0.15	0.7	1121.4
3	250	5000	0.2	0.8	1102.2
4	250	4000	0.2	0.7	1106.5
5	250	5000	0.2	0.6	1029.3
6	250	6000	0.2	0.7	989.1
7	250	6000	0.15	0.8	1088.8
8	250	5000	0.15	0.7	1133
9	300	5000	0.15	0.8	1206.1
10	300	4000	0.15	0.7	1211.1
11	300	6000	0.15	0.7	1103.7
12	300	5000	0.15	0.6	1143.2
13	250	6000	0.1	0.7	1099.9
14	200	5000	0.15	0.8	1084.2
15	200	5000	0.15	0.6	1001.9
16	250	4000	0.15	0.8	1189.1
17	250	5000	0.15	0.7	1107.7
18	250	4000	0.15	0.6	1102.3
19	200	5000	0.1	0.7	1095.5
20	250	6000	0.15	0.6	1005.2
21	300	5000	0.1	0.7	1207
22	250	5000	0.1	0.6	1139.2
23	250	4000	0.1	0.7	1206.5
24	250	5000	0.1	0.8	1182.3
25	200	6000	0.15	0.7	961.8
26	250	5000	0.15	0.7	1113.7
27	300	5000	0.2	0.7	1107.2
28	200	5000	0.2	0.7	985.9
29	250	5000	0.15	0.7	1159.1

**Table 4:** Analysis of variance of cutting depth regression model.

Source	Some of Squares	df	Mean Square	F-Value	*p*-Value Prob > F	
Model	1.402 × 10^5^	14	10,014.33	31.84	<0.0001	Significant
A-Laser power	57,463.68	1	57,463.68	182.73	<0.0001	
B-Feed speed	31,028.67	1	31,028.67	90.67	<0.0001	
C-Pulse frequency	28,577.28	1	28,577.28	98.87	<0.0001	
D-Water pressure	15,523.21	1	15,523.21	49.36	<0.0001	
AB	24.01	1	24.01	0.076	0.7863	
AC	640.09	1	640.09	2.04	0.1756	
AD	94.09	1	94.09	0.30	0.5930	
BC	29.16	1	29.16	0.093	0.7652	
BD	222.01	1	222.01	0.71	0.4149	
CD	2.56	1	2.56	8.140 × 10^−3^	0.9294	
A^2^	2949.31	1	2949.31	9.38	0.0084	
B^2^	216.20	1	216.20	0.69	0.4209	
C^2^	4649.59	1	4649.59	14.79	0.0018	
D^2^	53.55	1	53.55	0.17	0.6861	
Residual	4402.71	14	314.48			
Lack of Fit	2755.56	10	275.56	0.67	0.7243	Not significant
Pure Error	1647.15	4	411.79			
Cor Total	1.446 × 10^5^	28				
R-Squared = 0.9696			Adj R-Squared = 0.9391			

**Table 5:** The actual value after optimization is compared with the predicted value.

Laser Power (A)	Feed Speed (B)	Pulse Frequency (C)	Water Pressure (D)	Prediction Depth	Actual Depth	Error
W	mm/s	HZ	MPa	μm	μm	%
271.49	0.149	4048.24	0.799	1212.257	1189.97	1.84

## Data Availability

The datasets used or analyzed during the current study are available from the corresponding author on reasonable request.

## References

[1] Ou Y., Zhu D., Zhang H., Yao Y., Mobasher B., Huang L. (2016). Mechanical properties and failure characteristics of CFRP under intermediate strain rates and varying temperatures. Compos. Part B Eng..

[2] Geier N., Davim J.P., Szalay T. (2019). Advanced cutting tools and technologies for drilling carbon fibre reinforced polymer (CFRP) composites: A review. Compos. Part A Appl. Sci. Manuf..

[3] Oh S., Lee I., Park Y.-B., Ki H. (2019). Investigation of cut quality in fiber laser cutting of CFRP. Opt. Laser Technol..

[4] Zhang Y., Qiao H., Zhao J., Cao Z., Yu Y. (2020). Numerical simulation of water jet–guided laser micromachining of CFRP. Mater. Today Commun..

[5] Selzer R., Friedrich K. (1997). Mechanical properties and failure behaviour of carbon fibre-reinforced polymer composites under the influence of moisture. Compos. Part A Appl. Sci. Manuf..

[6] Xu W.-X., Zhang L.-C. (2015). Ultrasonic vibration-assisted machining: Principle, design and application. Adv. Manuf..

[7] Chen Y., Liang Y., Xu J., Hu A. (2018). Ultrasonic vibration assisted grinding of CFRP composites: Effect of fiber orientation and vibration velocity on grinding forces and surface quality. Int. J. Light. Mater. Manuf..

[8] George P.M., Raghunath B.K., Manocha L.M., Warrier A.M. (2004). EDM machining of carbon–carbon composite—A Taguchi approach. J. Mater. Process. Technol..

[9] Lau W.S., Wang M., Lee W.B. (1990). Electrical discharge machining of carbon fibre composite materials. Int. J. Mach. Tools Manuf..

[10] Freitag C., Wiedenmann M., Negel J.P., Loescher A., Onuseit V., Weber R., Ahmed M.A., Graf T. (2015). High-quality processing of CFRP with a 1.1-kW picosecond laser. Appl. Phys. A.

[11] Salama A., Li L., Mativenga P., Whitehead D. (2016). TEA CO_2_ laser machining of CFRP composite. Appl. Phys. A.

[12] Riveiro A., Quintero F., Lusquiños F., del Val J., Comesaña R., Boutinguiza M., Pou J. (2012). Experimental study on the CO_2_ laser cutting of carbon fiber reinforced plastic composite. Compos. Part A Appl. Sci. Manuf..

[13] Sander R., Poesl H., Frank F., Meister P., Strobel M., Spuhler A. (1988). An Nd:YAG laser with a water-guided laser beam—A new transmission system. Gastrointest. Endosc..

[14] Hock K., Adelmann B., Hellmann R. (2012). Comparative Study of Remote Fiber Laser and Water-Jet Guided Laser Cutting of Thin Metal Sheets. Phys. Procedia.

[15] Wagner F.R., Boillat C., Buchilly J.M., Spiegel A., Vago N., Richerzhagen B. (2003). High-speed cutting of thin materials with a Q-switched laser in a water-jet vs. conventional laser cutting with a free running laser. High-Power Lasers Appl..

[16] Perrottet D., Housh R., Richerzhagen B., Manley J. (2005). Heat damage-free Laser-Microjet cutting achieves highest die fracture strength. Photon Process. Microelectron. Photonics IV.

[17] Sun D., Han F., Ying W., Jin C. (2018). Surface integrity of water jet guided laser machining of CFRP. Procedia CIRP.

[18] Tabie V.M., Koranteng M.O., Yunus A., Kuuyine F. (2019). Water-Jet Guided Laser Cutting Technology—An Overview. Lasers Manuf. Mater. Process..

[19] Adalarasan R., Santhanakumar M. (2015). Application of Taguchi based Response Surface Method (TRSM) for Optimization of Multi Responses in Drilling Al/SiC/Al_2_O_3_ Hybrid Composite. J. Inst. Eng. (India) Ser. C.

[20] Freitag C., Kononenko T.V., Weber R., Konov V.I., Graf T. (2018). Influence of pulse repetition rate and pulse energy on the heat accumulation between subsequent laser pulses during laser processing of CFRP with ps pulses. Appl. Phys. A.

[21] Porter J.A., Louhisalmi Y.A., Karjalainen J.A., Füger S. (2007). Cutting thin sheet metal with a water jet guided laser using various cutting distances, feed speeds and angles of incidence. Int. J. Adv. Manuf. Technol..

